# Sterility

**Published:** 1897-03

**Authors:** 


**Sterility.** By Robert Bell, M.D. Pp. 88. London: J. & A. Churchill. 1896.

In his preface Dr. Bell has given us his views on the relative importance of the ovum and spermatozoa, but he has not advanced any proof which can upset the generally accepted ideas upon this subject. It is not a fact that the cause of sterility is very seldom on the part of the male, as the author would have us think. We have known a good many cases where the after-history of the women has proved that the fault of not bearing children did not rest with them.

We are disposed to admit that disease of the endometrium does account for a large number of women not bearing children, but that it is the main cause of sterility we very much doubt. Our own experience leads us to say that pregnancy does occur frequently in these women, but abortion at a very early stage often sets in, and these cases are put down as illustrative of true sterility. Of course the disease which produces early abortion is as fatal to the increase of population as those diseases which altogether prevent conception; but we are sure that a large number of women do become pregnant and go on to their full time with what the author would describe as endometritis, yet it is useful to bear in mind that this condition is one of the common causes of sterility, and one that can be remedied. It is well known that many abnormal conditions of the pelvic viscera lead to unhealthy states of the endometrium, and it is simply begging the question to say that these—the primal causes—are not the real sinners, but that the endometritis which they produce must be so regarded. Dr. Bell looks too much at one aspect of sterility, and overlooks a good many real and potent factors which have to be reckoned with. There is such a thing as relative fecundity, as was well laid down by Matthews Duncan, and we are convinced that many women perfectly normal in every respect do not conceive for reasons that we know very little about. The author's experience and our own are the same in so far that we recognise that a good many pelvic conditions such as he mentions do not give rise to symptoms until secondary changes take place in the endometrium; but we certainly have found sterility to exist in a large proportion of these cases long before symptoms of endometritis were complained of. The author's treatment of endometritis is of course perfectly sound, and is that adopted by most gynæcologists.

A good portion of the work goes beyond the scope of its title and really belongs more to the general subject of gynæcology. These little books, however, serve a very useful purpose by drawing attention to the views of men whose experience entitles them to write with authority.

